# EHMTI-0200. Aerobic exercise training at the ventilatory threshold prevents migraine and improves mood

**DOI:** 10.1186/1129-2377-15-S1-D45

**Published:** 2014-09-18

**Authors:** AB Oliveira, RT Ribeiro, MT Mello, S Tufik, MFP Peres

**Affiliations:** 1 Neurologia and Neurocirurgia, Universidade Federal de São Paulo, São Paulo, Brazil; 2 Psicobiologia, Universidade Federal de São Paulo, São Paulo, Brazil

## Introduction

The ventilatory threshold (VT) is a standardized, individual parameter of metabolic demand during exercise. No study on the preventive effect of aerobic exercise training (AE) for migraine (M) has used the VT.

## Aims

To measure changes in M clinical outcomes and mood state after a standardized AE program.

## Methods

The study recruited episodic M patients with and without aura (ICHDII) taking no preventive medicine. Study protocol comprised 12-week of AE, performed 3 times/wk, 30 min./session at the VT. Participants were randomly allocated for AE (EXE) or waiting list (CT) groups. VT was determined by a computerized open-circuit gas analyser during maximal cardiopulmonary exercise test for assessment of aerobic fitness (VO2max). The corresponding heart rate, workload, and perceived effort at VT were used to monitor AE intensity. Days with M (Days), M frequency (FREQ), disability (DIS), medication (MED), depression (BECKII), anxiety (GAD7) and mood (POMS) were measured at baseline and after AE.

## Results

25 patients (EXE, N = 13; CT, N = 12) completed the study. All participants' characteristics and baseline measurements matched between groups. CT changed no post-intervention variable. For EXE, there were reduction in DAYS (8.9±3.6 vs 5.6±3.4, p = 0.002), FREQ (6.3±3.0 vs 3.8±2.4, p = 0.002), POMS (27.3±35.1 vs 6.7±13.1, p = 0.038), GAD7 (7.2±5.1 vs 4.2±3.3, p = 0.034), and a trend to decrease in BECKII (6.3±3.0 vs 3.8±2.4, p = 0.067), and increase in VO2max (30.8±6.5 vs 32.1±5.5, p = 0.049) after AE.

## Conclusions

Metabolic-matched AE using VT prevented M and improved mood.

No conflict of interest.

